# Non-aneurysmal spontaneous subarachnoid hemorrhage: perimesencephalic versus non-perimesencephalic

**DOI:** 10.5935/0103-507X.20160028

**Published:** 2016

**Authors:** Luís Guilherme Bastos Silva Aguiar Coelho, José Manuel Dias Costa, Elsa Irene Peixoto Azevedo Silva

**Affiliations:** 1 Department of Neuroradiology, Centro Hospitalar de São João - Porto, Portugal.; 2 Department of Neurology, Centro Hospitalar de São João - Porto, Portugal.

**Keywords:** Tomography, x-ray computed, Vasospasm, intracranial, Subarachnoid hemorrhage, Hydrocephaly, Angiography, digital subtraction, Inpatients

## Abstract

**Objective:**

To compare the clinical evolution of perimesencephalic subarachnoid hemorrhage and non-perimesencephalic subarachnoid hemorrhage.

**Methods:**

The study was conducted retrospectively in a tertiary hospital center in the north region of Portugal. Included patients had no identifiable cause for subarachnoid hemorrhage. Several epidemiologic, clinical and imaging aspects were statistically analyzed, taking into account the differences in perimesencephalic subarachnoid hemorrhage and non-perimesencephalic subarachnoid hemorrhage.

**Results:**

Sixty-two patients met the inclusion criteria (46.8% - perimesencephalic subarachnoid hemorrhage; 53.2% - non-perimesencephalic subarachnoid hemorrhage). Demographic and clinical background characteristics were similar in both groups. Complications were more frequent in patients with non-perimesencephalic subarachnoid hemorrhage - 84.8% of the patients had at least one complication versus 48.3% in perimesencephalic subarachnoid hemorrhage. Vasospasm, infection and hydrocephaly were the most common complications (each was detected more frequently in the non-perimesencephalic subarachnoid hemorrhage group than in perimesencephalic subarachnoid hemorrhage group). Two patients died, both had a non-perimesencephalic subarachnoid hemorrhage. The median inpatient time was longer in the non-perimesencephalic subarachnoid hemorrhage group (21 versus 14 days). No incidents of rebleeding were reported during the follow-up period (mean time of 15 ± 10.3 months).

**Conclusion:**

Perimesencephalic subarachnoid hemorrhage and non-perimesencephalic subarachnoid hemorrhage are two different entities that have different clinical outcomes, namely in terms of complication rate and median inpatient time. The management of these patients should respect this difference to improve treatment and optimize health care resources.

## Abstract (pt)

**Objetivo:**

Comparar a evolução clínica da hemorragia subaracnóidea perimesencefálica com a da hemorragia subaracnóidea não perimesencefálica.

**Métodos:**

Estudo retrospectivo, que incluiu pacientes portadores de hemorragia subaracnóidea sem causa conhecida em um hospital terciário localizado na região norte de Portugal. Os dados epidemiológicos, clínicos e de imagem foram analisados estatisticamente, levando em conta a divisão dos pacientes em duas categorias: hemorragia subaracnóidea perimesencefálica e hemorragia subaracnóidea não perimesencefálica.

**Resultados:**

Cumpriram os critérios de inclusão 62 pacientes, 46,8% deles com hemorragia subaracnóidea perimesencefálica e 53,2% com hemorragia subaracnóidea não perimesencefálica. As caraterísticas demográficas, assim como os antecedentes clínicos, foram similares entre os grupos. As complicações foram observadas mais comumente no grupo com hemorragia subaracnóidea não perimesencefálica, sendo que 84,8% desses pacientes tiveram, no mínimo, uma complicação, comparados a 48,3% dos pacientes com hemorragia subaracnóidea perimesencefálica. Vasoespasmo, infecções e hidrocefalia foram as complicações mais comuns - todas observadas mais frequentemente nos pacientes com hemorragia subaracnóidea não perimesencefálica. Dois pacientes vieram a falecer, ambos com hemorragia subaracnóidea não perimesencefálica. A mediana do tempo de permanência no hospital foi maior nos pacientes com hemorragia subaracnóidea não perimesencefálica (21 dias, em comparação aos 14 dias observados nos pacientes com hemorragia subaracnóidea perimesencefálica). Não se observaram recidivas de sangramento durante o acompanhamento (tempo médio de 15 ± 10,3 meses).

**Conclusão:**

As hemorragias subaracnóideas perimesencefálica e não perimesencefálica tiveram formas diferentes de evolução clínica, principalmente no que se referiu à taxa de complicações e ao tempo mediano de permanência no hospital. Assim, a abordagem dessas duas formas de hemorragia subaracnóidea deve ser distinta, tanto em busca de melhorar o tratamento dos pacientes quanto para obter um melhor aproveitamento dos recursos de saúde.

## INTRODUCTION

Subarachnoid hemorrhage (SAH) refers to bleeding into the space between the arachnoid and pia-matter. It accounts for approximately 5% of strokes and occurs at a relatively young age.^(1)^

Generally, SAH occurs subsequent to the rupture of an aneurysm or of a vascular malformation, but in 15 to 20% of cases the cause remains unknown even after two or more angiographic studies^(2-4)^ (non-aneurysmal SAH). In 1985, van Gijn et al.^(4)^ subdivided this entity in two groups with different clinical outcomes. The division was made according to blood distribution observed during the initial cerebral computed tomography (CT), performed on first 24 hours after clinical ictus. Perimesencephalic SAH (PM-SAH) is characterized by blood in the perimesencephalic cisterns anterior to the brainstem that could extend to the *ambiens* cisterns and basal parts of the sylvian fissures; the non-perimesencephalic SAH (NPM-SAH) pattern has a more diffuse blood distribution that exceeds the previously mentioned regions.^(4,5)^

Generally, studies evaluating non-aneurysmal SAH describe a higher prevalence of PM-SAH, but this is not a universal finding and there are some studies indicating that NPM-SAH is more common.^(3,6,7)^ There are no published studies evaluating the prevalence and clinical outcome of patients with PM-SAH or NPM-SAH in Portugal.

The goal of this study was to evaluate the prevalence and clinical prognosis of patients with perimesencephalic subarachnoid hemorrhage and non-perimesencephalic subarachnoid hemorrhage.

## METHODS

This was a retrospective study that was approved by the Ethics Committee of *Centro Hospitalar de São João* (#232-15) and did not require informed consent. The current study encompassed a six year period in a tertiary hospital center in the northern region of Portugal. The patients included in the study had been discharged with a diagnosis of SAH of unidentifiable cause despite undergoing an angiographic study.

Patient data included the following: gender, age, clinical background, clinical presentation symptoms/signs, admission evaluation scales (Hunt & Hess (H&H), World Federation of Neurological Surgeons (WFNS) and Fisher) imaging studies, complications, length of hospital stay, patient assessment at follow-up appointments and Modified Rankin Scale (mRS) evaluation at three months.

PM-SAH and NPM-SAH groups were established according criteria defined by van Gijn et al.^(4)^ using an initial CT scan (within the first 24 hours of hospital admission).

The complications of vasospasm and hydrocephaly were considered present when clinical evidence was found and/or when diagnostic complementary exams documented complications; transcranial Doppler ultrasound criteria^(8)^ was used to examine vasospasm and imaging studies (usually CT) were used for hydrocephaly.

Overall outcome was evaluated using the mRS three months after hospital discharge.

Data were statistically analyzed with Statistical Package for Social Science (SPSS) version 20^(c)^: the chi square test was used for categorical variables, Student's *t* test was used to evaluate variables with a normal distribution (age, granted by the Kolmogorov-Smirnov and Shapiro-Wilk tests) and Mann-Whitney test was used to evaluate variables without a normal distribution (inpatient period). We considered differences to be statistically significant when the p value was lower than 0.05 and differences to have a statistically significant trend when the p value was between 0.05 and 0.08. Items without requisites for statistical analysis were presented descriptively.

## RESULTS

Sixty-five patients fulfilled inclusion criteria; three were excluded because the diagnosis of SAH was established without evidence of blood on the CT image (lumbar puncture) (n = 2) or patients had a convexity SAH pattern (n = 1), resulting in a final number of 62 patients. This number represented 18.1% of all spontaneous SAH cases admitted to our institution during the study period; 29 patients (46.7%) were diagnosed with PM-SAH and 33 patients (53.3%) were diagnosed with NPM-SAH.

Demographic characteristics (Table 1) were similar in both groups; no significant differences were found in terms of age (mean age PM-SAH 52.41 ± 11.76 years and NPM-SAH 56.82 ± 12.66 years) or gender (ratio male to female was 1:1.25 in both groups). Clinical background was also identical, except for the prevalence of diabetes mellitus, which was higher in the NPM-SAH group (p = 0.017).

**Table 1:** Demographic data

	**PM-SAH** ** (N = 29)**	**NPM-SAH** ** (N = 33)**	**Total**	**p value**
Age	52.4 ± 11.8	56.8 ± 12.7	54.76 ± 12.2	0.163
Gender				
Male	44.8 (13)	45.5 (15)	28	0.961
Female	55.2 (16)	54.5 (18)	34	
Dyslipidemia	37.9 (11)	42.4 (14)	25	0.719
Diabetes mellitus	10.3 (3)	37.4 (12)	15	0.017
Smoker/ex-smoker	34.5 (10)	24.2 (8)	18	0.375
Obesity	20.7 (6)	15.2 (5)	11	0.569
Stroke	6.9 (2)	3.0 (1)	3	0.479
Other	44.8 (13)	57.6 (19)	32	0.316

PM-SAH - perimesencephalic subarachnoid hemorrhage; NPM-SAH - non-perimesencephalic subarachnoid hemorrhage. Results are expressed as number (%) or as the mean ± standard deviation.

Headache was the most common symptom, reported by 100% of patients with PM-SAH and 90.9% of patients with NPM-SAH. Decreased consciousness was more common in patients with NPM-SAH, with a p value close to statistical relevance (0.055); no other symptom/sign was remarkably different between the two groups (Table 2). 

**Table 2:** Clinical presentation and admission scales

	**PM-SAH** ** (N = 29)**	**NPM-SAH** ** (N = 33)**	**Total**	**p value**
Headache	100 (29)	90.9 (30)	59	0.096
Vomiting/nausea	72.4 (21)	78.8 (26)	47	0.559
Decreased consciousness	17.2 (5)	39.4 (13)	18	0.055
Seizures	3.4 (1)	0.0 (0)	1	0.468
Nuchal stiffness	65.5 (19)	53.1 (17)	36	0.326
Hunt & Hess				
1	20.7 (6)	18.2 (6)	12	0.803
2	69.0 (20)	57.6 (19)	39	0.354
3	6.9 (2)	18.2 (6)	8	0.186
4	3.4 (1)	3.0 (1)	2	0.926
5	0.0 (0)	3.0 (1)	1	0.345
WFNS				
1	89.7 (26)	54.5 (18)	44	0.002
2	3.4 (1)	24.2 (8)	9	0.020
3	3.4 (1)	3.0 (1)	2	0.926
4	3.4 (1)	15.2 (5)	6	0.120
5	0.0 (0)	3.0 (1)	1	0.345
Fisher				
1	0.0 (0)	0.0 (0)	0	n/a
2	44.8 (13)	15.2 (5)	18	0.010
3	27.6 (8)	27.3 (9)	17	0.978
4	27.6 (8)	57.6 (19)	27	0.017

PM-SAH - perimesencephalic subarachnoid hemorrhage; NPM-SAH - nonperimesencephalic subarachnoid hemorrhage; WFNS - World Federation of Neurological Surgeons; n/a - non-aplicable. Results are expressed as number (%).

In terms of admission grading scales (Table 2), no significant differences were noted in the H&H scale score; patients in both groups exhibited a predominance for lower and more benign grades, mainly in the PM-SAH group (89.7% scored 1 or 2 in PM-SAH versus 75.7% in NPM-SAH). Similarly, according to the WFNS scale, the majority of patients in both groups scored 1 or 2 (93.1% of PM-SAH and 78.7% of NPM-SAH), but PM-SAH patients were more likely to have a score of grade 1 (89.7% versus 54.5%, p = 0.002). For Fisher's grade, no patients scored 1 (this grade is defined by the lack of blood detected on the CT scan); a score of 2 was calculated in 13 patients with PM-SAH (44.8%) versus in five patients with NMPM-SAH (15.2%) (p = 0.010), and a score of 4 was calculated in eight patients with PM-SAH (27.6%) versus 19 patients with NPM-SAH (57.6%) (p = 0.017).

Angiographic studies included CT angiography on admission for every patient. Digital subtraction angiography was performed subsequently in 91.9% (n = 57) of patients and 42.1% of these (n = 24) patients repeated the exam. Patients who did not undergo digital subtraction angiography (n = 5) were from the PM-SAH group: two patients were evaluated with repeated CT angiography, two patients underwent magnetic resonance imaging angiography and one patient did not require any additional exam.

Vasospasm, infection and hydrocephaly were the most common complications (n = 15 for the former two and n = 14 for the latter) in all patients. Incidents of rebleeding were not reported.

Complications (Table 3) were more common in patients with NPM-SAH; 84.8% of patients experienced at least one complication, versus 48.3% of patients in the PM-SAH group (p = 0.02), representing a 6-fold increased complication risk in patients in the NPM-SAH group. Every complication was more common in the NPM-SAH group. This difference was statistically significant in regards to hydrocephaly (p = 0.031); patients with NPM-SAH exhibited a 4.3 times higher risk of developing hydrocephaly. Differences in the occurrence of vasospasm or infections exhibited a trend without statistical significance (p = 0.073).

**Table 3:** Complications

	**PM-SAH** ** (N = 29)**	**NPM-SAH** ** (N = 33)**	**Total**	**p value**
Vasospasm	13.8 (4)	33.3 (11)	24.5 (15)	0.073
Ischemia	0.0 (0)	6.1 (2)	3.2 (2)	0.279 (Fisher)
Rebleeding	0.0 (0)	0.0 (0)	0.0 (0)	n/a
Hydrocephaly	10.3 (3)	33.3 (11)	22.6 (14)	0.031
Seizures	3.4 (1)	12.1 (4)	8.1 (5)	0.220 (Fisher)
Hyponatremia	17.2 (5)	18.2 (6)	17.7 (11)	0.923
Infections	13.8 (4)	33.3 (11)	24.5 (15)	0.073
Other	20.7 (6)	33.3 (11)	27.4 (17)	0.265
Any complication	48.3 (14)	84.8 (28)	67.7 (42)	0.002

PM-SAH - perimesencephalic subarachnoid hemorrhage; NPM-SAH - nonperimesencephalic subarachnoid hemorrhage; n/a - non-applicable. Results are expressed as number (%).

The median inpatient period was 14 days in the PM-SAH group (50% of cases between 12 and 16.5 days) and 21 days in the NPM-SAH group (50% of cases between 15 and 28 days). This difference was statistically significant (p < 0.001).

Two patients died during the hospital stay due to infectious complications (cerebral ventriculitis and pneumonia); both patients had been diagnosed with NPM-SAH (mortality rate of 7.1%).

Forty-nine patients (82% of the surviving patients) had follow-up appointments in our institution. The remaining 11 were lost to follow-up, as they had been transferred to other centers. The mean time of follow-up was 15.8 ± 10.3 months; no incidents of rebleeding were reported during this period. Excellent recovery (mRS 0 - 1) at three months after hospital discharge was found in 95.7% of patients diagnosed with PM-SAH and 85.7% of patients diagnosed with NPM-SAH (difference statistically irrelevant, p = 0.235).

## DISCUSSION

This study confirms that spontaneous SAH is not a homogeneous disease. PM-SAH and NPM-SAH are distinct diseases with differing clinical progressions that commonly fall under the same designation, non-aneurysmal SAH. NPM-SAH exhibits a more aggressive clinical course, with a higher rate of complications and a longer hospital stay.^(9)^ The length of hospital stay is an aspect that was often neglected in previous studies evaluating non-aneurysmal SAH, but it is of crucial importance due to inherent personal and economic costs.

Globally, our findings are in line with previous international studies, although in Portugal, information regarding this issue was lacking.

We reported a higher number of females in both groups, similar to Ildan et al.^(10)^ and contrary to the majority of previous studies that reported a slight male predominance.^(6,7)^

Diabetes mellitus^(8)^ is not recognized as a major risk factor for SAH,^(11)^ but has been related to a higher risk of vasospasm.^(12)^ We did not observe that diabetes was associated with vasospasm in the current study.

As expected, patients diagnosed with NPM-SAH had higher Fisher scale scores^(13)^ and a higher risk of vasospasm,^(14,15)^ which is in line with notion that patients with an aggressive clinical presentation and a greater amount of blood detected in the subarachnoid space have a poorer prognosis.^(16)^

In the current study, the incidence of vasospasm in the PM-SAH and NPM-SAH groups was higher than in the majority of other studies.^(6,7,13,17,18)^ This difference may be explained by the fact that we considered the existence of vasospasm when transcranial Doppler ultrasound blood flow velocity criteria were met and not just when clinical vasospasm was observed, which is in contrast to previous studies. Transcranial Doppler ultrasound is the recommended method for monitoring SAH patients for the development of vasospasm^(19)^ and it has been proven to be accurate.^(20)^ Hydrocephaly was also detected more often in our study compared to previous investigations. We identified the presence of hydrocephaly when an imaging study documented it at any time during the inpatient period,^(1)^ whereas in other studies hydrocephaly was recorded only when permanent. We decided to report these complications as positive, even when no clinical signs were observed, because these patients have a different clinical approach (type of unity care, medical care supervision), which could possibly interfere with the final clinical outcome. Nevertheless, the difference in hydrocephaly incidence between the two groups (higher in NPM-SAH) is similar to the results reported in other studies.^(7,13,21)^

In spite of the differences in clinical progression between the two groups, patient outcomes at three months generally indicated a good prognosis both for NPM-SAH patients and PM-SAH patients. Our findings suggest that even though NPM-SAH exhibits a more aggressive clinical presentation, patients with NPM-SAH had a better prognosis compared to patients with aneurysmal SAH.^(22)^

Patients with convexity SAH were not included in the study, because this disease has a different pathophysiology and clinical evolution than non-aneurysmal SAH.^(23-26)^

Patients in whom a diagnosis of SAH was made based on a positive lumbar puncture alone were not included in the study, the SAH pattern could not be determined.

Current evidence suggests that angiographic studies in non-aneurysmal SAH patients should include two digital subtraction angiography, especially in patients diagnosed with NPM-SAH.^(6,27)^ In our institution, this decision was made on a case by case basis by a multidisciplinary medical team. The analysis showed a decision pattern whereby most patients with NPM-SAH underwent a repeated digital subtraction angiography; patients with PM-SAH were evaluated with different approaches, although the majority of them underwent a digital subtraction angiography study after a negative angiographic CT. The relatively long time of follow-up without reports of rebleeding is a good indicator of the negative predictive value of this decision pattern. A specific analysis of the decision pattern was not a purpose of our study, although this type of analysis would be an interesting aim for a larger study.

The limitations of this study include the fact that this is a retrospective analysis, the size of the cohort is small, the absence of two digital subtraction angiographies performed in every patient and the limited data from follow-up appointments.

## CONCLUSION

The perimesencephalic subarachnoid hemorrhage and non-perimesencephalic subarachnoid hemorrhage are two different diseases with different clinical evolutions. Non-perimesencephalic subarachnoid hemorrhage has a more diffuse subarachnoid blood distribution, a more aggressive clinical presentation and a higher probability of complications.

Non-perimesencephalic subarachnoid hemorrhage has a longer inpatient period with higher economical costs for health care systems. In non-aneurysmal subarachnoid hemorrhage, the initial computed tomography scan might have therefore impact in patient's management, prognosis, inpatient period, outcome and economical costs for healthcare systems.
